# Noncommunicable Disease (NCD) strategic plans in low- and lower-middle income Sub-Saharan Africa: framing and policy response

**DOI:** 10.1080/16549716.2020.1805165

**Published:** 2020-09-02

**Authors:** Chantelle Boudreaux, Christopher Noble, Matthew M. Coates, Jason Kelley, Martin Abanda, Alexander Kintu, Amy McLaughlin, Andrew Marx, Gene Bukhman

**Affiliations:** a Program in Global NCDs and Social Change, Department of Global Health and Social Medicine, Harvard Medical School, Boston, MA, USA; b Division of Global Health Equity, Department of Medicine, Brigham and Women’s Hospital, Boston, MA, USA; c NCD Synergies Project, Partners in Health, Boston, MA, USA; d Division of Cardiovascular Medicine, Department of Medicine, Brigham and Women’s Hospital, Boston, MA, USA; e Department of Global Health, Harvard T.H. Chan School of Public Health, Boston, MA, USA

**Keywords:** Cancer, cardiovascular disease, CVD, diabetes, chronic respiratory disease, CRD, behavioural risk factors, Global Action Plan, strategic plan, policy, Africa, Sub-Saharan Africa, low and lower-middle income countries

## Abstract

**Background:**

Global efforts to address NCDs focus primarily on 4-by-4 interventions – interventions to prevent and treat four groups of conditions affecting mainly older adults (some cardiovascular disease and cancers, type 2 diabetes, chronic respiratory disease) and four associated risk factors (alcohol, tobacco, poor diets, and physical inactivity). However, the NCD burden in Sub-Saharan Africa (SSA) is composed of a more diverse set of conditions, driven by a more complex group of risks, and impacting all segments of the population.

**Objective:**

To document the NCD priorities identified by NCD strategic plans, to characterize the proposed policy response, and to assess the alignment between the two.

**Methods:**

Using a two-part conceptual framework, we undertook a descriptive study to characterize the framing and overall policy response of strategic plans from 24 low- and lower-middle-income countries across SSA.

**Results:**

The national situation assessments that ground strategic plans emphasize a diversity of conditions that range in terms of severity and frequency. These assessments also highlight a wide diversity of factors that shape this burden. Most include discussions of a broad range of behavioral, structural, genetic, and infectious risk factors. Plans endorse a more narrow response to this diverse burden, with a focus on primary and secondary prevention that is generally convergent with the objectives established in global policy documents.

**Conclusions:**

Broadly, we observe that plans developed by countries in SSA recognize the heterogeneity of the NCD burden in this region. However, they emphasize interventions that are consistent with global strategies focused on preventing a narrower set of cardiometabolic risk factors and their associated diseases. In comparison, relatively few countries detail plans to prevent, treat, and palliate the full scope of the needs they identify. There is a need for increased support for bottom-up planning efforts to address local priorities.

## Background

Noncommunicable diseases (NCDs) have long posed significant challenges to health systems across Sub-Saharan Africa. While they often are thought of as a byproduct of growing wealth and aging populations, NCD rates have been high historically and are declining across the region [1]. At the same time, these conditions affect all segments of society, with diseases such as sickle cell disorder and rheumatic heart disease having a particularly high burden among impoverished and younger populations [2,3].

However, the visibility of NCDs has risen in the wake of the epidemiological transition in the region, which is characterized by significant progress in addressing communicable disease and maternal and child health [4]. As a consequence, the proportional burden of poor health attributed to NCDs continues to grow. This process is exacerbated by the demographic and social changes mentioned above, as well as increasing exposure to behavioral and metabolic risks [5]. As a result, even as the total disability-adjusted life years (DALYs) per person attributed to NCDs has been declining in Sub-Saharan Africa for the past 20 years, the share of total DALYs that is attributable to NCDs rose from 21% in 2007 to nearly 30% in 2017 [1]. This trend is likely to continue in the coming decades [6,7].

In response to this evolving disease burden, the World Health Assembly endorsed its first global strategy on NCDs in 2000 and released its first Global Action Plan in 2008 [8,9]. Since that time, there have been several high-profile efforts to organize the global response to this group of diseases. After being left off of the Millennium Development Goals, both the Sustainable Development Goals (SDGs) and the Universal Health Coverage (UHC) agenda include NCDs as a priority. More targeted efforts include multiple High-Level Meetings on the Prevention and Control of NCDs hosted by the United Nations and WHO’s publication of its second Global Action Plan for the Prevention and Control of NCDs in 2013 [10–13].

Much global energy has coalesced around preventable conditions, with a focus on four major disease groups that primarily affect older adults. These include some cancers, cardiovascular disease (CVD) including ischemic heart disease and stroke, type 2 diabetes, and chronic respiratory disease (CRD). These four conditions are frequently linked to four behavioral risk factors (tobacco use, sedentary lifestyles, poor diet, and the harmful use of alcohol) [9]. The so-called ‘4-by-4’ approach builds upon lessons learned from addressing cardiovascular and other chronic diseases in high-income countries [14]. It seeks to reduce NCD-linked morbidity and mortality by concentrating on primary and secondary prevention, including behavior change to avoid illness and early detection and treatment to avoid the most significant morbidity [15]. More recently, at the third High-Level Meeting on the prevention and control of NCDs, stakeholders from around the world agreed to expand this framework to include mental health and air pollution (‘5-by-5’) [12].

While momentum to address NCDs was initially slow to build in low- and lower-middle-income countries (LLMICs), there has been significant progress since the WHO’s first Global Action Plan called upon countries to establish national policies targeting these conditions in 2008. WHO’s NCD monitoring reports show that the percentage of countries in the African region with an ‘Operational, integrated policy, strategy or action plan’ increased from 17% in 2010 to 37% in 2013 and 72% in 2015 [16]. National NCD strategic plans are one such type of plan. They provide a framework to coordinate multisectoral action and, as such, play a critical role in achieving UHC in Africa [17]. They are both a vehicle to adapt these global priorities to local needs and a window into how countries articulate and frame national policy agendas. To date, however, there has been no systematic assessment of the existing domestic NCD policy landscape.

## Methods

### Eligibility Criteria

Inclusion criteria included (1) location in Sub-Saharan Africa, (2) low- or lower-middle-income status (LLMICs), and (3) the use of English or French languages for public policy documents. Upper-middle and high-income countries were excluded from the analysis. Relative to LLMICs, these countries face important differences in terms of service availability and resource constraints but were too small in number to allow for a stand-alone analysis.

### Information Sources and Search

NCD Strategic Plans were collected from three known publicly available repositories of strategic plans: the WHO Country Planning Cycle Database, the WHO NCD Document Repository, and the International Cancer Control Partnership (ICCP) National Plan Portal [18–20]. For the WHO Country Planning Cycle Database, we searched for any NCD plan by manually searching the repositories of all eligible countries in Africa. For the WHO NCD Document Repository, we searched for ‘Integrated NCD policies’ and selected for all eligible countries in Africa. For the ICCP, we selected for all countries in the Africa/Middle Africa, Africa/Eastern Africa, and Africa/Western Africa regions, and selected for NCD Plans.

### Document Selection

All documents for countries meeting these criteria were downloaded from the three above mentioned sources and reviewed to assess eligibility. Documents other than NCD strategic plans, including disease or risk factor specific strategic plans, were excluded from the complete review. For countries with multiple strategic plans, only the most recent document was reviewed. Each of these repositories was last reviewed in November 2019.

### Framework Development

For this descriptive study, we conducted a framework analysis using a deductive two-part conceptual framework [21]. Framework analysis offers a structured approach to map information onto a thematic matrix. The first part of the framework is used to document how countries present their local disease and risk burdens. The second part of the framework is used to understand how countries respond to this burden.

#### Defining Policy Priorities

The first part of the conceptual framework was designed to understand how countries describe local NCD priorities to which the subsequent policy proposals respond. Disease and risk categories were initially based on the 190 noncommunicable diseases and the 63 lowest-level risk factors included in the most detailed level of the Global Burden of Disease (GBD) 2015 Cause Hierarchy and were updated to reflect subsequent iterations of the Hierarchy from newer GBD studies, as well as to allow for the inclusion of conditions that could not be matched to the GBD [22]. A description of the process used to develop this framework is found in Appendix 1.

#### Defining the Policy Response

The second part of the framework was developed to understand how countries plan to respond to the local disease burden. Interventions were categorized according to several criteria, including the type of activity (policy, training, care provision) and the level of the health system that is targeted. Disease response efforts were grouped to prevention (e.g. education or vaccination), screening and diagnosis, and clinical management or treatment. Finally, given the role of the WHO’s Global Action Plans in promoting the development of strategic plans, the study team also documented the degree to which policies reflect the 2008–2013 and 2013–2020 Global Action Plans [9,13,23].

### Data Extraction

Using the conceptual frameworks described above, the research team reviewed and extracted data from each strategic plan. For each plan, the study team separately characterized the content of national situation assessments, policy plans, and monitoring and evaluation (M&E) plans. Data from M&E plans were captured separately because indicators included in monitoring frameworks often receive special attention during implementation.

In extracting data for the national situation assessment, reviewers indicated when the national strategic plan identified a given condition or risk factor as a specific concern within the country’s context. Descriptions of the global or regional disease contexts were not recorded, nor were definitional listings of disease categories. Risk factors were noted when a risk factor was both causally linked to any NCD in the document and locally contextualized. Data were captured separately by two reviewers, with reviewers first reviewing documents and agreeing upon relevant sections for detailed review. Cases of discordance in data entry were jointly reviewed and discussed by the two researchers for reconciliation. When agreement could not be achieved, a third reviewer was called to provide a final judgment.

The frameworks were modified over time to better reflect the conditions reported within national plans. Rows were added or deleted to reflect the disease, risk, and policy profiles presented by the documents. After reviewing the initial findings, a single researcher conducted a final review of all documents to supplement findings and clarify any questions raised during the initial analysis.

### Characterizing the NCD Priorities and Policy Response

After data extraction was complete, the study team undertook two steps to process the data. In the first, we grouped conditions into three major categories: (1) conditions that fall into the 4-by-4 framework – that is, conditions that fall within the four major disease categories and are associated with the four identified behavioral risk factors (Category 1); (2) conditions that generally fall into the four major disease categories but are not significantly associated with the four behavioral risk factors (Category 2); and (3) conditions that fall outside of the 4-by-4 group altogether (Category 3).

These categories were selected to allow for differences in the efforts needed to prevent and treat specific conditions. For example, efforts to strengthen clinical care for Category 1 conditions will often overlap with those in Category 2. However, Category 2 conditions will not be responsive to prevention campaigns targeting the modifiable risk factors that are of interest for Category 1 conditions. Category 3 conditions are unlikely to be impacted by either prevention campaigns or health systems strengthening efforts targeting Category 1 conditions. Table 1 provides an overview of this categorization framework, as well as examples of conditions falling into each category. Many conditions may fall within multiple categories: for example, liver cancer is associated with both alcohol consumption (Category 1) and with hepatitis B or C infection (Category 2).

**Table 1.** Overview of the disease categorization framework.

Category	Description
Category 1	Conditions falling into the four major disease groups (Cancer, Diabetes, Cardiovascular Disease, and Chronic Respiratory Disease) that are associated^a^ with the four prioritized risk factors (alcohol, tobacco, exercise, and diet) – e.g. “4-by-4 conditions.” Examples include ischemic heart disease, lung and esophageal cancer, type 2 diabetes, and chronic obstructive pulmonary disease.
Category 2	Conditions falling into the four major disease groups but that are linked to a broader set of risks including, for example, environmental, infectious, genetic, and idiopathic factors [5]. Examples include cervical cancer and rheumatic heart disease (of infectious origin), type 1 diabetes (of a combination of infectious, environmental, and genetic origin), and asthma (of environmental and genetic origin).
Category 3	Conditions falling outside of the four major disease groups. Examples include epilepsy, Noma, injuries, mental health conditions, and conditions affecting the sense organs.

^a^Defined as at least 10% population attributable fraction (PAF), using the attributable risk for Sub-Saharan Africa as identified in the Global Burden of Disease Project [5]. See Appendix 2 for additional information.

The second data processing step was taken to manually link policy proposals onto individual conditions. This step was taken when disease targets were not specifically stated. Through this process, both risk factor mitigation efforts and specific clinical interventions were mapped to individual diseases. Given the multiple routes through which the most common risk factors impact health, many such campaigns are attributed to multiple conditions. Potential impacts documented by the team may have been unanticipated by the policymakers.

Details on the processes used to define categories and to link map activities onto individual conditions can both be found in Appendix 2. Briefly, both steps rely on the population attributable fraction (PAF) – the proportion of the burden of a particular disease in a given population that could be averted through reduction in exposure to a risk factor to a minimum-risk level. We classified NCD conditions with at least 10% of their burden attributable to 4-by-4 risk factors within Category 1. Similarly, risk factor mitigation efforts were linked to diseases if the combination of activities targeted by the policy proposals jointly account for a minimum of 10% of the burden. Clinical interventions were mapped on to particular conditions in consultation with medical providers on the research team.

This 10% PAF cut-off was selected to allow for the complex relationship between behavior change campaigns and disease incidence [24]. With at least 10% of the burden measured in DALYs causally linked to some combination of the four behavioral risk factors, it is plausible to anticipate a change in disease incidence stemming from behavior change campaigns. The selection of a 10% cut-point follows cut-offs used elsewhere [25], but alternatives (12%, 15%) were also assessed with little qualitative impact on the findings. A description of the process used to calculate PAFs can be found in Appendix 2, as can PAFs for the range of conditions considered in this paper.

## Results

**Figure 1.**
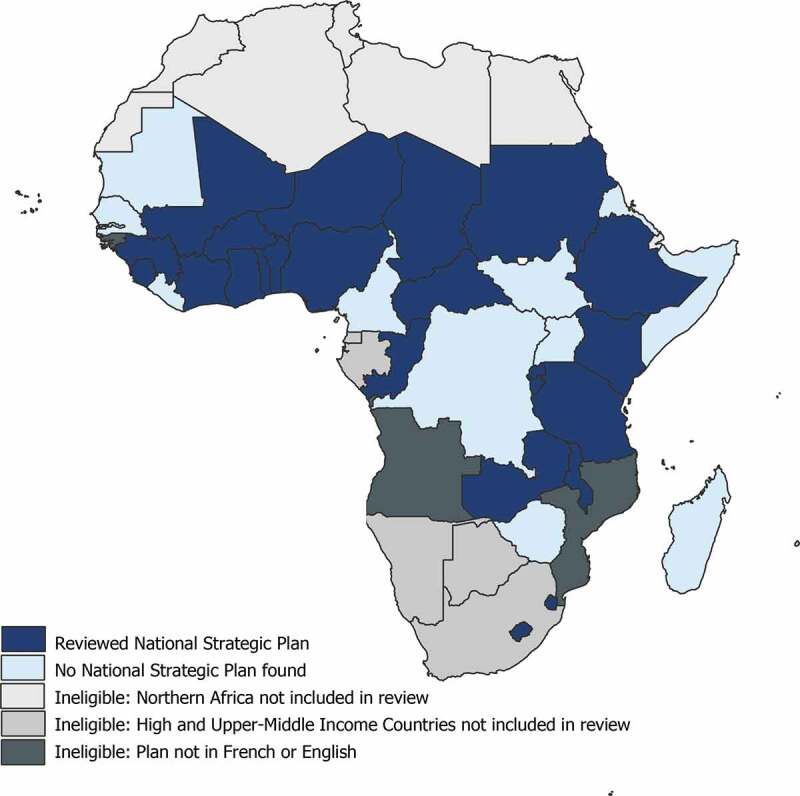
Map of NCD strategic plan availability.

Thirty-six countries met the inclusion criteria. For these countries, 24 eligible plans were identified and reviewed. These represented 52% of countries in Sub-Saharan Africa, and 67% of countries meeting the inclusion criteria. The largest single source of policy documents was the WHO’s NCD Document Repository, which collects all such plans as a part of its periodic Country Capacity Survey, last completed in May 2019 [19]. See Figure 1 for an overview of the inclusion status of countries across the region. The 12 countries for which strategic plans were not found are dispersed geographically and economically but were more often defined as fragile states by the UNDP, compared to countries with strategic plans. See Appendix 3 for complete details on the eligibility status of countries and details on the plans that were reviewed.

In dark blue, 24 countries were included in this review. Countries in shades of blue were eligible for inclusion in this study. Strategic plans were identified and reviewed for countries in dark blue. No strategic plan was identified for countries in light blue. Countries in shades of grey were ineligible for review, due either to language (darkest grey), income status (middle-grey) or location (lightest grey).

Of the 24 plans reviewed, all documents included a narrative situation assessment of the national NCD burden and either a written or detailed tabular (e.g. a work plan) policy response, and 15 documents were found to contain an M&E plan.

### Defining policy priorities

All of the strategic plans reviewed for this analysis included a narrative situation assessment to define the priorities for subsequent policy action. Discussions of the local burden of cancers, cardiovascular disease, and diabetes were present in all plans, and only one plan omitted a discussion of respiratory disease. At the same time, the countries rarely distinguish between Category 1 and Category 2 conditions. Countries frequently articulated broad links between the modifiable risk factors, such as tobacco use, and the local cancer and cardiovascular disease burdens. All plans identified at least one Category 3 condition. In particular, many countries reference mental health and neurological conditions, such as epilepsy, depressive and anxiety disorders, alcohol use disorders, and schizophrenia and other psychotic disorders. Injuries, including road traffic accidents and interpersonal violence, were also mentioned frequently. Sickle cell disease and road traffic accidents are the most widely documented Category 3 conditions. See Table 2 for a list of the most commonly mentioned conditions.

**Table 2.** Disease burdens described by national situation assessments within NCD strategic plans; N = 24 plans.

Category 1, Category 2, and Mixed Category 1 and 2	
**Cancer**	100%
Esophageal cancer	25%
Liver cancer	58%
Colon and rectum cancer	29%
Cervical cancer	83%
Prostate cancer	67%
Non-Hodgkin lymphoma	42%
Bladder cancer	21%
Ovary	21%
Nephroblastoma (Wilms Tumour)	21%
Skin Cancers	29%
Kaposi’s Sarcoma	38%
Breast cancer	79%
**Cardiovascular diseases**	100%
Hypertension	96%
Rheumatic heart disease	29%
Ischemic heart disease	54%
Stroke	63%
**Diabetes**	100%
**Chronic respiratory diseases**	88%
COPD	42%
Asthma	63%
**Category 3**	
Chronic kidney disease	46%
Sickle cell disorders	67%
Oral disorders	46%
Vision disorders, including Blindness/Cataract/Myopia	54%
**Mental, neurological and substance abuse**	79%
Epilepsy	58%
Depressive and Anxiety Disorders	42%
Schizophrenia and Other Psychotic Disorders	29%
Alcohol use disorders	33%
Drug use disorders	29%
**Injuries (Any)**	75%
RTA	67%
Falls	21%
Burns/Fire, heat, and hot substances	29%
Self-harm and interpersonal violence	50%

Similarly, nearly all countries described the large and growing importance of the four modifiable risk factors of diet, exercise, tobacco, and alcohol in their strategic plans. Only one country omitted a discussion of the roles of diet and tobacco in the local epidemiology of NCDs. All but two countries discussed the roles of exercise and alcohol abuse. As in the description of local NCD burdens, countries supplemented the discussion of risks with a review of a complex array of additional risks. These included environmental, structural, demographic, and infectious risks for NCDs, as well as behavioral factors falling outside of the 4-by-4 framing. Commonly cited infectious risks include HPV, HIV, and Hepatitis B. More than one-third of countries described the importance of NCD co-morbidity as a risk factor (e.g. describing causal relationships between cardiovascular disease and mental health, or between diabetes and chronic kidney disease). Indoor and outdoor air pollution, rapid urbanization, and poor road infrastructure were the most commonly cited environmental risks. Socioeconomic conditions, cultural norms, and regulatory frameworks were frequently cited as structural risk factors. See Table 3 for a list of the most commonly mentioned risk factors.

**Table 3.** Risk factors highlighted by national situation assessments within NCD strategic plans; N = 24 plans.

Risk factors	
**Any Behavioral Risk**	100%
Exercise/Sedentary Lifestyle	92%
Low fruit/vegetable/high fat, salt, excessive Diet	96%
Alcohol Abuse	92%
Smoking	96%
Drug Abuse/Khat	25%
Undernutrition/Micronutrient deficiency	25%
Unhealthy sexual behaviour	13%
Occupational	33%
**Any Infectious Risk or Comorbidity**	63%
HIV	38%
HPV	33%
Hepatitis B ± Hepatitis C	25%
Streptococcal for RHD	13%
NCD infection co-morbidity as risk factor (Depression and CVD; Diabetes and CKD)	38%
Other (Measles, H pylori, vaccine-preventable, neurocystocosis, trachoma)	17%
**Any Environmental Risk**	67%
Urbanization (Including difficulty exercising, overcrowding, stress)	29%
Traffic/Unsafe driving/Poor road infrastructure	29%
Outdoor Air Pollution	42%
Indoor Air pollution	33%
Pesticides	17%
Sun Exposure	13%
**Any Intermediate Risk**	96%
High Blood Pressure/Hypertension	92%
Obesity	96%
Raised blood lipid/Cholesterol	63%
High blood sugar/Hyperglycemia	54%
**Any Structural Risk**	38%
Cultural (Early Marriage/Communal living and Cooking/Traditional Food Preparation)	13%
Societal (education, employment and work options)	17%
Globalization	17%
Political/Regulatory	13%
Conflict/War/Violence	17%
Socioeconomic (Poverty, Unemployment)	25%
**Any Genetic or Biologic Risk**	50%
Genetic/Family history	46%
Sickle Cell	25%
Race	21%
Gender	25%
Ageing	42%

### Defining the policy response

Governments outline a range of actions to respond to local disease burdens. Proposed activities cover the entirety of the health sector. They include advocacy and policy development at the national level, strengthening of diagnosis and treatment at the facility level, and health education and promotion activities at the community level. Here, we consider risk factor mitigation and disease prevention, screening, and treatment separately.

#### Risk Factor Mitigation

**Figure 2.**
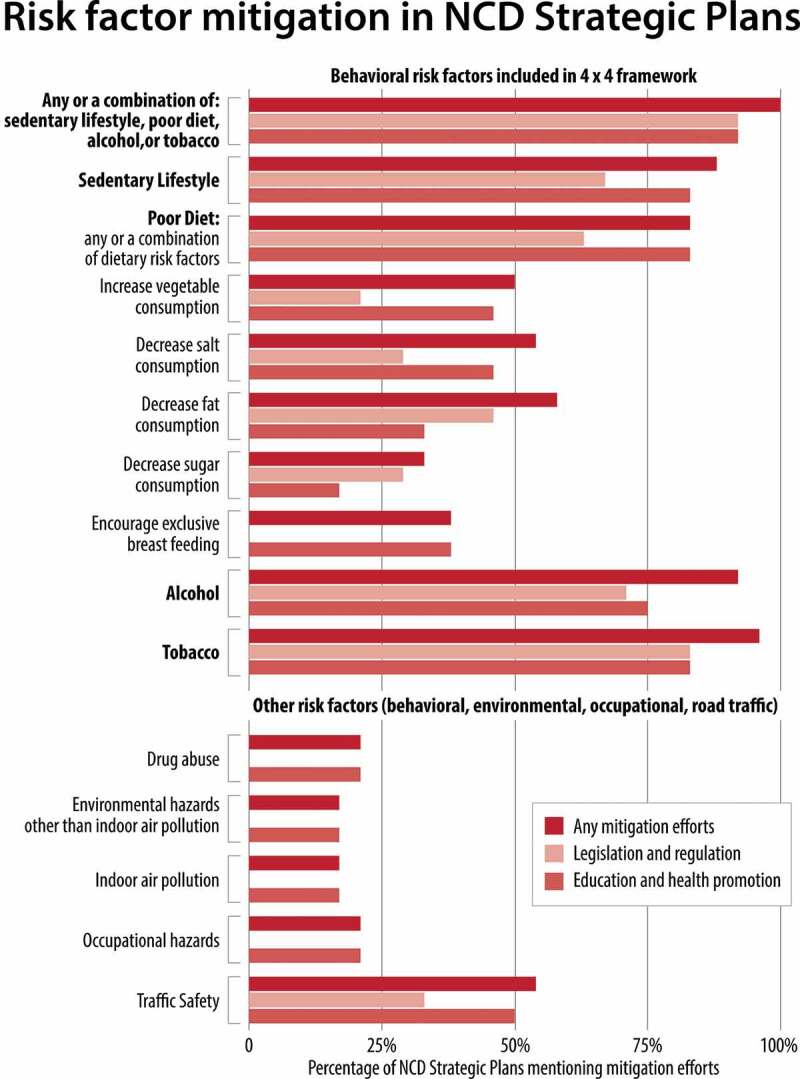
Risk factors targeted by education & promotion, legislation, or any mitigation efforts described in NCD strategic plans; N = 24 plans.

Efforts to mitigate modifiable risks include a broad array of potential interventions, and we identified nearly 100 distinct activities listed by countries. We divided these into (1) legislation and regulation and (2) education and health promotion campaigns. Figure 2 illustrates the relative frequency of legislative and education campaigns for a range of risk factors. All countries listed at least one action aimed at reducing exposure to alcohol, tobacco, sedentary lifestyles, or poor diets.

Education and promotion campaigns take a number of forms. Although many health promotion activities were focused on the community-level, broad social marketing campaigns and education efforts based out of health facilities were also common. All but four countries specified plans to educate the public on exercise, diet, and tobacco, and three-quarters specified education campaigns aimed at alcohol consumption.

Legislative and regulatory efforts mirrored these proposed campaigns. The range of policy tools varied by risk factor, but financial levers were among the most commonly cited policy tools. Two-thirds of countries described efforts to introduce or strengthen existing sin taxes on tobacco, alcohol, or sugary/fatty foods. Outside of the four behavioral risk factors, a quarter of countries propose legislation around traffic and injuries, particularly regarding road safety and driving conditions. Complete details on the use of regulatory and legislative actions proposed to address behavioral risk factors can be found in Table 4.

**Table 4.** Regulatory and legislative action proposed by NCD Strategic Plans to address behavioral risk factors; N = 24 plans.

**Any regulatory or legislative action targeting sedentary lifestyles**	67%
Encouraging built spaces for exercise	54%
Policies to encourage exercise in life, including schools and workplaces. E.g, no-car days, improved sidewalks	54%
Financial incentives to promote activity	4%
**Any regulatory or legislative action targeting diet**	63%
Minimizing/Eliminating trans/saturated fats in diet	46%
Reducing salt content	29%
Requirements re: diet and or food content	25%
Mandates re: marketing and labeling of foods	25%
Food labeling requirements	25%
Restrictions on marketing of sugar	25%
Subsidies on fruits and vegetables	21%
Guidelines on diet in the workplace, schools	21%
Reducing sugar content	17%
Sin taxes on Sugar or Fat	13%
Encouraging local food production	8%
**Any regulatory or legislative action tobacco use**	83%
Sin Taxes on tobacco	63%
Restrictions on tobacco advertising	58%
Smoke-Free spaces	54%
Restrictions on tobacco sales	38%
Warning labels requirements for tobacco products	29%
Creation or strengthening of tobacco import permits	25%
Registration requirements for tobacco companies	13%
Promote alternative livelihoods for tobacco farmers	8%
**Any regulatory or legislative action alcohol use**	71%
Sin taxes on alcohol	46%
Restrictions on production or marketing of alcohol	42%
Implement or enforce drunk driving laws	38%
Restrictions on the sale of alcohol	38%
Restrictions on alcohol advertising	33%
Implement or enforce legal age limits for alcohol consumption	21%
Regulate alcohol importation	13%
Create alcohol-free spaces	13%
Mandate alcohol labels	4%

##### Disease prevention, diagnosis, and treatment

**Figure 3.**
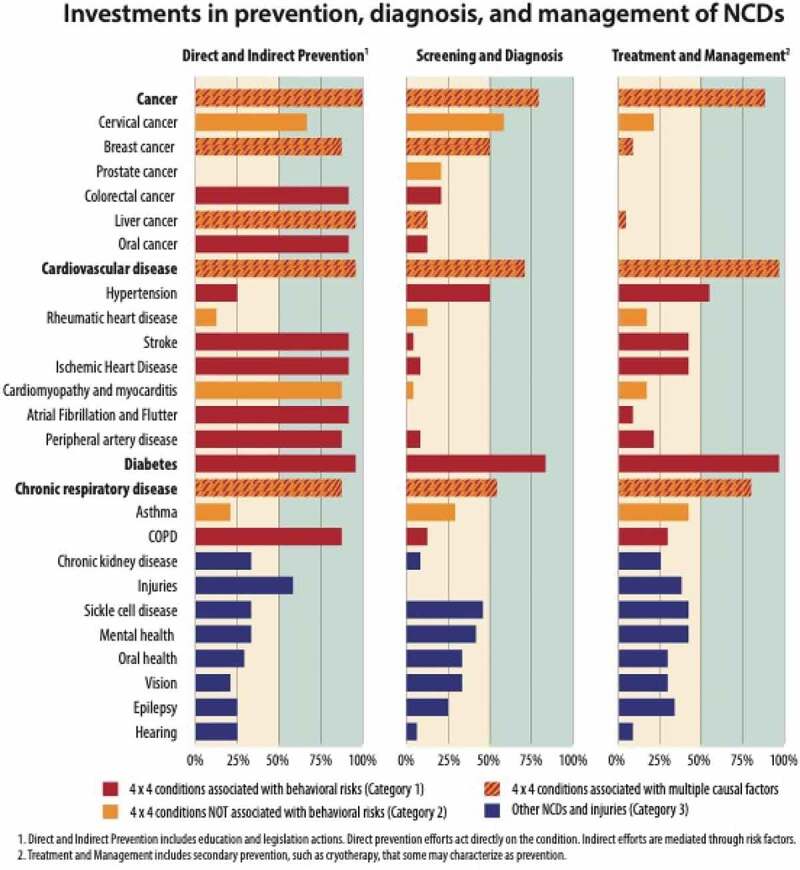
Conditions affected by activities specified by NCD strategic plans; N = 24 plans.

Figure 3 provides an overview of the distribution of prevention, diagnosis, and treatment efforts described by strategic plans. The first panel highlights the conditions impacted by direct and indirect prevention efforts. Direct channels include disease-specific education or vaccination campaigns, while indirect channels represent the expected downstream impact of the risk factor education campaigns highlighted above. The second panel highlights targeted efforts to expand access to screening and diagnosis for the specified conditions. The third panel highlights plans to strengthen clinical treatment and management. Figure 3 is designed to assess the potential impact of activities. It documents interventions that act directly and indirectly, intentionally or not.

All countries describe prevention campaigns. These include both primary prevention, such as vaccination campaigns for HPV and hepatitis B, and secondary prevention, such as treatment of streptococcal infections to prevent rheumatic heart disease. Education programs targeting a combination of behavioral risk factors and conditions explain the high prevalence of prevention programs outside of vaccine-preventable conditions.

Many countries describe plans to expand access to screening and early detection of disease. Community screening for diabetes is the most common, followed by chronic respiratory disease and hypertension. Countries also describe efforts to improve early detection of cancers, including cervical cancer, breast cancer, prostate cancer, and colorectal cancer. Beyond the four major NCDs, countries also describe efforts to screen for sickle cell disease, eye health, oral health, and epilepsy. Plans to provide treatment options generally aligned with efforts to expand screening, although there were exceptions. Efforts to screen for cervical cancer, for example, outpace efforts to provide treatment by nearly three times. Screenings for breast cancer outpace treatment by six times.

Countries describe a wide variety of activities aimed at treating and managing these conditions. All but four countries described plans to improve service readiness and availability at the facility level, mainly through increasing access to drugs and equipment and improvements in infrastructure. Countries also identified human resource constraints as an important challenge to the achievement of outlined activities, and all but two strategic plans outlined training plans. Training efforts emphasized cadres working in primary care and communities, and tasks related to prevention, screening, and diagnosis. While most countries focus on training that can be delivered to existing health workers, some also describe alternative strategies to strengthen human resources, including efforts to increase the overall number of workers and task-shifting.

A majority of countries explicitly framed their national strategies in relation to global policy documents. Seventeen of the 24 plans referred to the Political Declaration of the 2011 General Assembly on the Prevention and Control of NCDs. The same proportion referred to either the 2008 or 2013 versions of the WHO’s Global Action Plan for the Prevention and Control of NCDs. Regional policy documents were also prominent; half of the plans referenced the 2011 WHO African Region Ministerial Consultation on Noncommunicable Disease or the subsequent Brazzaville Declaration. A closer review of the alignment between the strategic plans and the objectives laid out by the Global Action Plan can be found in Appendix 4.

### Mapping priorities on to action

We found that situation assessments considered a more expansive set of conditions than did policy response or M&E sections of the plans. For example, the 15 countries with all three sections of the plans listed an average of 20 distinct conditions in the situation assessment, 13 conditions in the policy response, and eight conditions in the M&E.

**Figure 4.**
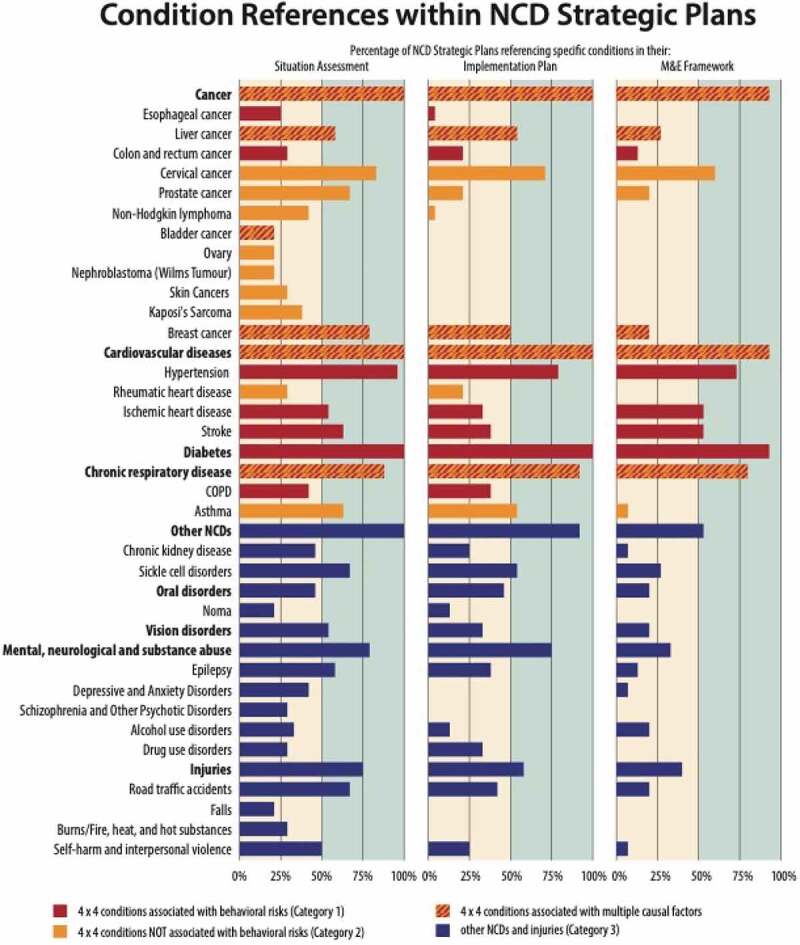
Condition references within NCD strategic plans: N = 24 plans for Panel 1: Situation Assessment and Panel 2: Policy Response; N = 15 plans for Panel 3: M&E.

The types of diseases mentioned are also notably different between sections. Policy responses and M&E plans aligned more closely with the 4-by-4 framework established by global policy documents. Consider the following Category 1 conditions: type 2 diabetes is mentioned in all situation assessments, in all policy responses, and in but one of the M&E plans; hypertension follows a similar pattern. By contrast, consider a prominent Category 2 condition. Rheumatic heart disease is highlighted in fewer than one-third situation assessments, one-fifth of policy responses, and no monitoring frameworks. Epilepsy, a prominent Category 3 condition, provides a parallel case study. While it is highlighted in over half of national situation assessments, it features in two of the fifteen M&E plans. Figure 4 provides an overview of this phenomenon for the broader spectrum of conditions considered by at least 20% of countries.

Note the distinction between Figures 3 and 4. Figure 4 is designed to understand what conditions countries emphasize in their plans. It tracks specific references to individual conditions across the various sections of the document. By contrast, Figure 3 is designed to assess the potential impact of activities, and documents interventions that act directly and indirectly, intentionally or not.

## Discussion

This analysis illustrates a sizable gap between the perceived drivers of local NCD burdens across Sub-Saharan Africa – as indicated by the national situation assessments – and the policy actions that countries take to address the NCD burden. Relative to situation assessments, implementation plans are more limited in both the overall number and diversity of conditions. Policy responses tend to converge on the four diseases and risk factors prioritized in global documents. Because ‘what gets measured gets done,’ the critical importance of measurement tools has long been recognized [26]. Our review finds that the convergence on the four-by-four conditions and global policy documents is even more evident in M&E plans, with locally notable conditions such as rheumatic heart disease and Noma completely absent from monitoring indicators.

The policy documents, thus, fail to directly respond to many of the conditions prioritized at the outset. A closer analysis of the policy response allows us to assess not only what is prioritized but how countries respond to the issues identified. We find an emphasis on primary and secondary prevention efforts, many of which require multisectoral action and collaboration. This is particularly the case for the various cancers and cardiovascular diseases, diabetes, and chronic respiratory diseases that comprise Category 1 and Category 2 conditions. Regulatory and legislative action is required for the introduction and enforcement of new rules around tobacco and alcohol production, marketing, distribution, and use. Industry engagement is needed for widespread changes to food content, and the participation of municipalities and institutions is necessary to introduce improvements to road- and walkways [27,28]. More than a simple coordination problem, many of the regulations face active opposition from the tobacco, alcohol, and food industries, whose economic interests are threatened [29–31]. They can also stimulate opposition from within the government when they come into other government priorities, including economic and trade targets [32].

While we identify an emphasis on prevention over treatment in the policy plans, this may not translate cleanly into action on the ground. Researchers in Malawi found that the implementation of community-based prevention activities lagged behind facility-based treatment efforts [33]. This is despite the known challenges facing NCD care delivery in the region and may link to the challenges associated with implementing multisectoral action described above [34]. Researchers have documented weak political will and limited technical capacity of local NCD units as challenges in engaging and coordinating stakeholders from outside of the health sector in national NCD response efforts [32]. Limited engagement in both formulation and implementation, in turn, has been linked to delays or failures of the resulting policies [35].

This study has several limitations. Methodologically, we rely on GBD data to develop our disease category framework. Data limitations within the GBD may result in imprecise or incorrect relationships, particularly for less common risk factors. More broadly, we applied relatively strict inclusion criteria to this review, including language and income restrictions, and a clear focus on the integrated strategic plans. While these decisions help to ensure comparability across documents, policy information may have been missed. As a result, we can say little about variations in policy planning across income groups or between anglophone and francophone countries. Lusophone countries were not included in this review. Nor can we draw clear distinctions between countries with and without strategic plans. While we identify few clear trends across a range of indicators assessed, countries without plans may differ in other important ways from those that were included in this review.

Finally, this work does not examine the process underlying the development of strategic plans. With the growing visibility of NCDs, the last decade has seen a dramatic expansion in the number of countries developing NCD strategic plans in Africa [8]. Prior case studies have documented a heavy reliance on global policies including the WHO’s Global Action Plan for the Prevention and Control of NCDs, which may account for our findings [31,35,36]. While global guidance has been credited with the expansion in national plans, it does not guarantee success. And, as others have noted, the existence of well-articulated documents has not necessarily lead to fully implemented policies [8,31,37]. Indeed, despite the close alignment between the global and domestic policy documents, research suggests slow overall progress in the implementation of the most recent Global Action Plan in Africa [38].

Researchers elsewhere have argued that this slow progress reflects a failure to adequately localize the national response, due to gaps in local data and limited involvement of stakeholders outside of the health sector [32,35,39,40]. Others have identified a poor alignment between NCD-specific and sector-wide plans in terms of goals, financial resource allocation, and implementation details [8]. NCD plans are often developed by technical teams at the MOH, with little participation of sector-wide policy or planning units. NCD units often lack financial resources and technical capacity needed to implement or monitor their plans. They rely heavily on nongovernmental organizations in the absence of strong governance or coordination structures [31,32].

While our analysis does not speak directly to these issues, it does point to potentially important signposts along the way. The situation assessments developed by countries are intended to establish the epidemiologic and health systems challenges to which the subsequent policy proposals respond. While the resulting policy decisions must inevitably accept trade-offs across a range of competing priorities – including global priorities – a failure to respond to pressing local issues can undermine local buy-in for a process that is fraught with political and coordination challenges. More work is needed to assess the implementation status of these NCD policies, as well as the impact of these policies on disease prevention and control across the region.

## Conclusion

We observe that strategic plans developed by countries in sub-Saharan African recognize the heterogeneity of the NCD burden in this region. However, policy agendas emphasize interventions that are consistent with global plans focused on preventing a narrower set of cardiometabolic risk factors and their associated diseases [36]. As a result, there is frequently a misalignment between the analysis of the situation and the policy prescriptions. This misalignment has the potential to leave gaps in national NCD policies and send conflicting messages to donors regarding financing needs.

Consequently, we find that there is a need for strategic plans in Sub-Saharan Africa that are based on systematic priority-setting grounded in local information and needs. Strengthening NCD policy in sub-Saharan Africa will require increased support for government NCD divisions and WHO country and regional offices. This finding is consistent with prior calls for a broadening of the NCD agenda in this region [3].

## Figures and Tables

**Figure A4.**
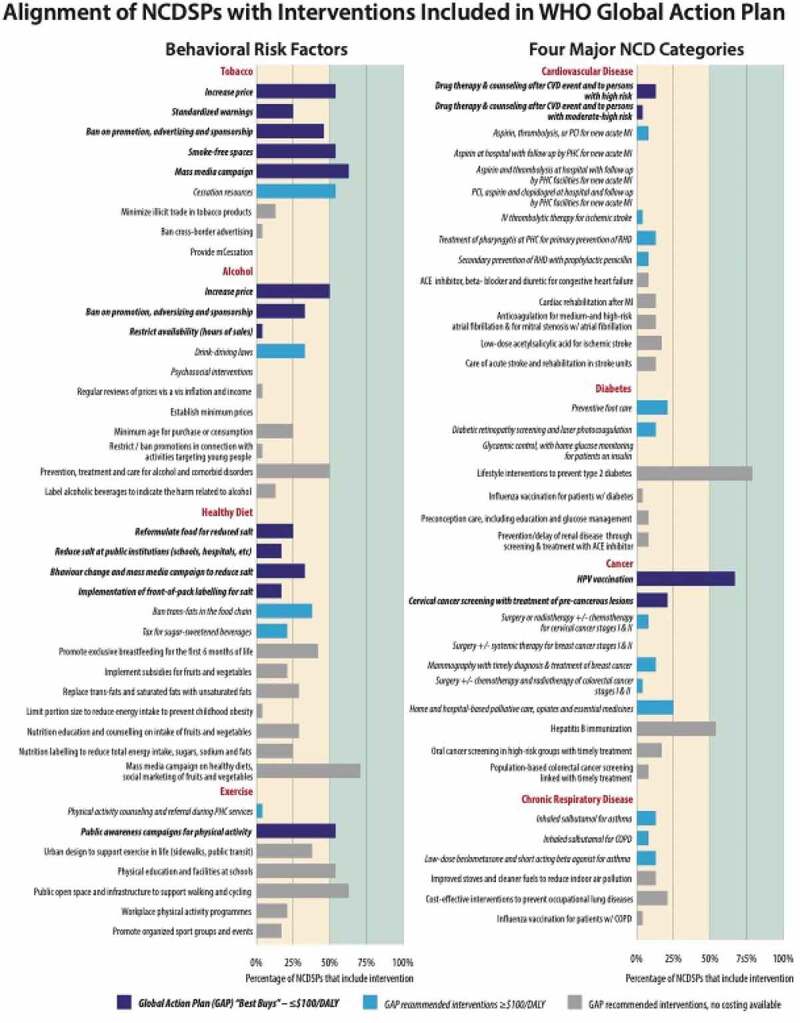
Frequency of WHO “Best Buys” and other Global Action Plan activities in the NCD Strategic Plans; n=24.

Global Action Plan Objective 3, targeting risk factors, (left) and Objective 4, strengthening health systems for prevention and control of NCDs, (right). Dark blue interventions are WHO “Best Buys,” having been identified as very cost- effective (cost-effectiveness ratio (CER) of ≤$100/DALY). Lighter blue interventions are less cost-effective, CER >$100/DALY. Remaining interventions had not been assessed for cost-effectiveness at the time that the guidance was published [23].

## Paper context

Strategic Plans play an important role in adapting global recommendations to local NCD burdens and priorities. We provide a descriptive policy review of NCD strategic plans in Sub-Saharan Africa. Broadly, we observe that strategic plans developed by countries in SSA recognize the heterogeneity of the NCD burden in this region. However, they emphasize interventions that are consistent with global strategies focused on preventing a narrower set of cardiometabolic risk factors and their associated diseases. In comparison, relatively few countries detail plans to prevent, treat, and palliate the full scope of the needs they identify. There is a need for increased support for bottom-up planning efforts to address local epidemiology and priorities.

## Appendix 1. Development of the disease framework

Given the length of the GBD cause hierarchy, the research team sought to develop a concise, but comprehensive, list of the disease and risks to be included in the full review. In an effort to achieve this list, the survey team employed an iterative process of manual and automated text analysis on a databank of 29 NCD policy documents from countries across the world. The list of documents included some of plans included in this study, as well as documents from outside of the study region (predominately documents from Asia), and older plans from the study region that since been updated. The specific steps taken are as follows:

- An initial list of NCD and Injury disease and risk categories was compiled using then current (2015) GBD cause hierarchy list.
- Using Medical Subject Headings (MeSH), this list was expanded to include synonyms for the different disease and risk factors.
- The resulting list of search terms was tested for comprehensiveness using an iterative process of manual and automated accounting of term mentions within the aforementioned group of policy documents. Automated review relied on the text mining package ‘tm’ in R [41,42].
- Any term identified at least once via the automated step was included in initial iteration of the framework.
- From this framework, additional rows were added as they were identified in the manual text review. In post-review, these terms were usually found to fit within the GBD hierarchy and were placed accordingly.

## Appendix 2. Calculation of population attributable fraction and linking indirect prevention and treatment efforts to individual conditions

The PAF reflects a combination of susceptibility to the risk factor, degree of additional risk of disease conveyed by exposure to the risk factor, and the prevalence of the exposure. Because the prevalence of risk factors varies geographically, the PAF also varies by location. For this analysis, we used the combined PAF across the 24 included countries. To determine the proportion of burden from a given cause attributable to the four major risk factors, we calculated joint attribution to the set of risks from the GBD Study 2017 that includes alcohol use, tobacco use, low physical activity, and a number of dietary risks. We followed the GBD methodology to jointly calculate risks, which makes a simplifying assumption that risks are independent in the population [5]. The general formula is shown below, where PAF_ri_ is the PAF for risk i and is combined for a set of risks r_1_-r_n_.

(1) $$PAF_{r_{1}-r_{n}}=1-\prod_{i=1}^{n}\left(1-PAF_{r_{i}}\right)$$

The effects of some of the risk factors we included were mediated through other risks (e.g. diet low in legumes through diet low in fibre). This mediation is incorporated in calculations to avoid double counting. In the formula below, PAF_r1-rn_ is the population attributable fraction for the given risk or set of risks, r1-rn, and MF is the mediation factor, representing the proportion of attribution for risk that is mediated through risk j, ri/rj. We used mediation factors estimated in the GBD Study [5].

(2) $$PAF_{r_{1}-r_{n}}=1-\prod_{i=1}^{n}(1-PAF_{r_{i}}*(\prod_{j=1}^{m}\left(1-MF_{\frac{r_{i}}{r_{j}}}\right)))$$

- PAFs for the conditions considered in this review are provided in Table 2A below.

**Table 2A.** PAFs for the conditions most commonly included in strategic plans.

Condition	Population attributable fraction (% of total)
Alcohol use disorders	99.97
Ischemic heart disease	79.94
Stroke	61.77
Esophageal cancer	56.64
Cardiovascular diseases	54.55
Diabetes mellitus	36.83
Liver cancer	24.51
Chronic obstructive pulmonary disease	21.61
Neoplasms	14.40
Breast cancer	13.74
Epilepsy	12.70
Chronic respiratory diseases	12.52
Self-harm and interpersonal violence	11.95
Road injuries	6.97
Injuries	6.43
Cervical cancer	3.98
Fire, heat, and hot substances	3.66
Asthma	3.44
Prostate cancer	3.27
Chronic kidney disease	2.91
Rheumatic heart disease	1.52
Blindness and vision impairment	0.92
Anxiety disorders	Unquantified
Depressive disorders	Unquantified
Drug use disorders	Unquantified
Malignant skin melanoma	Unquantified
Mental disorders	Unquantified
Non-Hodgkin lymphoma	Unquantified
Non-melanoma skin cancer	Unquantified
Oral disorders	Unquantified
Schizophrenia	Unquantified
Sickle cell disorders	Unquantified

The GBD is subject to data and resource constraints that are of note in this analysis. Data underlying the analyses may be incomplete or incorrect. In this case, models are likely to mischaracterize given relationships. GBD may also lack the information needed to model a given relationship. When this occurs, 0% of the risk is attributed to a condition. From the information available, it is not possible to distinguish between a relationship that is truly zero, and a relationship that has not been reviewed.

In particular, a number of PAFs in the database are listed as ‘0.’ For example, although there is likely to be some causal relationship between mental health conditions and alcohol abuse, the PAF for the four risk factors is zero. Similarly, although we expect some relationship between diet and oral disorders, the PAF for the four risk factors is also zero. To avoid mischaracterizing such relationships in the table above, we refer to all such findings as ‘Unquantified.’

*Linking the Policy Response to Individual Diseases and Risk Factors:* Strategic plans describe activities that impact disease prevention and management, both directly and indirectly. We defined activities as direct if the plan explicitly links an action to a particular condition, while we defined activities as indirect if there was no explicit link in the plan but there is a clear mechanism through which the activity addresses the burden. Direct impacts stem from activities that aim to strengthen prevention, diagnosis, or management of identified conditions. Examples include plans to implement community-based screening for diabetes or to build a stroke unit at a tertiary hospital. In either case, the condition targeted is clearly specified.

Indirect activities can be divided into disease prevention, generally through risk factor mitigation, and therapeutic management of a disease. While a clear link exists between the activity and a given condition, this link is not made explicitly within the plan. Indirect therapeutic interventions were attributed to particular conditions in consultation with clinicians. Indirect prevention efforts were credited if a plan targets a combination of risk factors that jointly account for at least 10% of the disease burden.

Linking indirect prevention campaigns to individual conditions

The GBD calculates country-specific, regional and global PAFs for particular risks based on local risk factor prevalence estimates. For this analysis, we used the higher of the regional or global estimate. For each country, we combine the PAFs for the joint set of risks featured within a policy plan. An example of the resulting matrix is shown in Table 2B, below. In each case, a country was credited with prevention campaigns if the joint PAF was greater than 10%. These indirect efforts were combined with disease-specific prevention campaigns (e.g. HPV vaccination campaigns to prevent cervical cancer) to generate the results presented in the main text.

Characterization of Therapeutics

Clinicians on the research team were consulted to link particular medicines to one or more specific conditions. In particular, efforts to expand access to aspirin and statins were classified as treatment for ischemic heart disease, cardiomyopathy, stroke, and peripheral artery disease. Efforts to expand access to beta blockers are classified as treatment for ischemic heart disease and cardiomyopathy, and efforts to expand access to anticoagulants are classified as treatment for atrial fibrillation and flutter.

**Table 2B.** PAF of the risk factors addressed, by country.

Prevention via risk factor mitigation, PAF of the risk factors mentioned																								
	Benin	Burkina Faso	Burundi	Central African republic	Chad	Comoros	Congo	Cote d’Ivoire	Ethiopia	Ghana	Guinea	Kenya	Lesotho	Malawi	Mali	Niger	Nigeria	Rwanda	Sierra Leone	Sudan	Swaziland	Tanzania	Togo	Zambia
Neoplasms	35	0	35	35	40	36	35	37	36	35	35	38	35	35	0	35	40	35	35	35	15	36	34	35
Cervical cancer	10	0	10	10	10	10	10	10	10	10	10	10	10	10	0	10	10	10	10	10	0	10	10	10
Breast cancer	16	0	16	16	16	16	16	16	16	16	16	16	16	16	0	16	16	16	16	16	12	16	7	16
Prostate cancer	7	0	7	7	7	7	7	7	7	7	7	7	7	7	0	7	7	7	7	7	0	7	7	7
Colon and rectum cancer	52	0	52	52	52	52	52	52	52	52	52	52	52	52	0	52	52	52	52	52	44	52	43	52
Liver cancer	34	0	34	34	44	44	34	34	44	34	34	34	34	34	0	34	44	34	34	34	20	34	30	34
Lip and oral cavity cancer	71	0	71	71	71	71	71	71	71	71	71	71	71	71	0	71	71	71	71	71	46	71	50	71
Cardiovascular diseases	70	0	70	70	75	70	70	73	70	70	70	72	70	70	0	70	75	70	70	70	61	73	71	70
Rheumatic heart disease	0	0	0	6	8	0	0	6	6	6	0	8	6	0	0	0	8	0	0	0	6	0	8	6
Non-rheumatic valvular heart disease	0	0	0	4	6	0	0	4	4	4	0	6	4	0	0	0	6	0	0	0	4	0	6	4
Stroke	70	0	70	70	76	70	70	74	70	70	70	73	70	70	0	70	76	70	70	70	62	74	70	70
Ischemic heart disease	83	0	83	83	88	83	83	86	83	83	83	85	83	83	0	83	88	83	83	83	76	86	85	83
Cardiomyopathy and myocarditis	29	0	29	32	33	29	29	32	32	32	29	33	32	29	0	29	33	29	29	29	32	29	5	32
Atrial fibrillation and flutter	20	0	20	28	30	20	20	28	28	28	20	30	28	20	0	20	30	20	20	20	22	20	21	28
Peripheral artery disease	33	0	33	37	38	33	33	37	37	37	33	38	37	33	0	33	38	33	33	33	6	33	38	37
Diabetes mellitus	47	0	47	47	59	47	47	54	47	47	47	52	47	47	0	47	59	47	47	47	37	54	51	47
Chronic respiratory diseases	31	0	31	31	56	31	31	48	31	31	31	49	31	31	0	31	56	31	31	31	0	40	49	31
Asthma	9	0	9	9	18	9	9	18	9	9	9	18	9	9	0	9	18	9	9	9	0	9	18	9
Chronic obstructive pulmo0ry disease	40	0	40	40	69	40	40	62	40	40	40	58	40	40	0	40	69	40	40	40	0	56	58	40
Chronic kidney disease	0	0	0	10	13	0	0	10	10	10	0	13	10	0	0	0	13	0	0	0	10	0	13	10
Injuries	10	0	10	10	19	11	10	18	11	10	10	18	10	10	0	10	19	10	10	10	10	10	10	10
Mental disorders	0	0	0	0	2	0	0	0	0	0	0	2	0	0	0	0	2	0	0	0	0	0	2	0
Blindness and vision impairment	2	0	2	2	9	2	2	9	2	2	2	2	2	2	0	2	9	2	2	2	0	9	2	2
Epilepsy	15	0	15	15	15	15	15	15	15	15	15	15	15	15	0	15	15	15	15	15	15	15	0	15
Age-related and other hearing loss	0	0	0	0	21	0	0	21	0	0	0	21	0	0	0	0	21	0	0	0	0	0	21	0

## Appendix 3. Eligibility status and plan selection of ncd strategic plans

**Table 3A.** Eligibility/inclusion status of countries.

Country name	Eligibility status
Algeria	Not eligible, Outside of SSA
Angola	Not eligible, Language
Benin	Eligible and Complete
Botswana	Not eligible, Income
Burkina Faso	Eligible and Complete
Burundi	Eligible and Complete
Cabo Verde	Not eligible, Language
Cameroon	Eligible, but strategic plan not found
Central African Republic	Eligible and Complete
Chad	Eligible and Complete
Comoros	Eligible and Complete
Congo	Eligible and Complete
Côte d’Ivoire	Eligible and Complete
Democratic Republic of the Congo	Eligible, but strategic plan not found
Djibouti	Not eligible, Outside of SSA
Egypt	Not eligible, Outside of SSA
Equatorial Guinea	Not eligible, Income
Eritrea	Eligible, but strategic plan not found
Eswatini/Swaziland	Eligible and Complete
Ethiopia	Eligible and Complete
Gabon	Not eligible, Income
Gambia	Eligible, but strategic plan not found
Ghana	Eligible and Complete
Guinea	Eligible and Complete
Guinea-Bissau	Not eligible, Language
Kenya	Eligible and Complete
Lesotho	Eligible and Complete
Liberia	Eligible, but strategic plan not found
Libya	Not eligible, Outside of SSA
Madagascar	Eligible, but strategic plan not found
Malawi	Eligible and Complete
Mali	Eligible and Complete
Mauritania	Eligible, but strategic plan not found
Mauritius	Not eligible, Income
Morocco	Not eligible, Outside of SSA
Mozambique	Not eligible, Language
Namibia	Not eligible, Income
Niger	Eligible and Complete
Nigeria	Eligible and Complete
Rwanda	Eligible and Complete
São Tomé and Príncipe	Not eligible, Language
Senegal	Eligible, but strategic plan not found
Seychelles	Not eligible, Income
Sierra Leone	Eligible and Complete
Somalia	Eligible, but strategic plan not found
South Africa	Not eligible, Income
South Sudan	Eligible, but strategic plan not found
Sudan	Eligible and Complete
Togo	Eligible and Complete
Tunisia	Not eligible, Outside of SSA
Uganda	Eligible, but strategic plan not found
United Republic of Tanzania	Eligible and Complete
Zambia	Eligible and Complete
Zimbabwe	Eligible, but strategic plan not found

**Table 3B.** NCD strategic plans included in this review.

Country	Plan	Year	Situation assessment	Policy response	M&E
Benin	Plan Stratégique Intégré de Lutte Contre les Maladies Non-Transmissibles	2014–2018	Yes	Narrative Response and Workplan	Yes
Burkina Faso	Plan Stratégique Intégré de Lutte Contre les Maladies Non-Transmissibles	2016–2020	Yes	Narrative Response and Workplan	Yes
Burundi	Plan Stratégique Intégré de Lutte Contre les Maladies Chroniques Non-Transmissibles	2011–2015	Yes	Narrative Response only	No
Central African Republic	Plan pour la lutte contre les maladies non transmissibles 2015–2021	2015–2021	Yes	Narrative Response and Workplan	Yes
Chad	Plan Multisectoriel de Lutte et de Contrôle des Maladies Non Transmissibles	2017–2021	Yes	Narrative Response and Workplan	Yes
Comoros	Document de stratégie nationale de prévention et de lutte contre les maladies non transmissibles; Draft 1	2013	Yes	Narrative Response and Workplan	No
Congo	Plan National Intégré de Lutte Contre Les Malades Non-Transmissibles au Congo	2013–2017	Yes	Narrative Response only	No
Cote D’Ivoire	Plan Stratégique Intégré de Prévention et de Prise en Charge des Maladies Non Transmissibles en Côte d’Ivoire	2015–2019	Yes	Narrative Response and Workplan	Yes
Ethiopia	National Strategic Action Plan (NSAP) for Prevention & Control of Non-Communicable Diseases in Ethiopia	2014–2016	Yes	Narrative Response and Workplan	No
Ghana	Strategy for the Management, Prevention and Control of Chronic Non- Communicable Diseases in Ghana	2012–2016	Yes	Narrative Response and Workplan	Yes
Guinea	Programme National Intégré de Prévention et de Contrôle des Maladies Non Transmissibles	2011–2015	Yes	Narrative Response only	Yes
Kenya	Kenya National Strategy for the Prevention and Control of Non-Communicable Diseases	2015–2020	Yes	Narrative Response and Workplan	Yes
Lesotho	National Multi-Sectoral Integrated Strategic Plan for Prevention and Control of Non-communicable Diseases	2014–2020	Yes	Narrative Response and Workplan	Yes
Malawi	National Action Plan for the Prevention and Management of Non-Communicable Diseases in Malawi	2012–2016	Yes	Narrative Response and Workplan	No
Mali	Plan Stratégique National de Lutte Contre Les Maladies Non Transmissibles	2015–2019	Yes	Narrative Response and Workplan	No
Niger	Plan Stratégique National de Prévention et de Lutte contre les MNT; Draft Amélioré	2012	Yes	Narrative Response and Workplan	No
Nigeria	National Policy and Strategic Plan of Action on Prevention and Control of Non-Communicable Diseases (NCDs)	2013	Yes	Narrative Response and Workplan	No
Rwanda	Rwanda Non-Communicable Diseases and Injuries National Strategic Plan	2014–2019	Yes	Narrative Response and Workplan	Yes
Sierra Leone	National Non-Communicable Diseases Strategic Plan	2013–2017	Yes	Narrative Response and Workplan	Yes
Sudan	Non-communicable Disease National Strategic Plan	2010–2015	Yes	Narrative Response and Workplan	No
eSwatini (Swaziland)	Swaziland National NCDs Strategic Plan; Draft	2012–2020	Yes	Narrative Response and Workplan	Yes
Tanzania	Strategic Plan and Action Plan For The Prevention And Control Of Non Communicable Diseases In Tanzania	2016–2020	Yes	Narrative Response and Workplan	Yes
Togo	Politique et Plan Stratégique Intégré de Lutte Contre les Maladies Non Transmissibles	2012–2015	Yes	Narrative Response and Workplan	Yes
Zambia	Zambian Strategic Plan 2013–2016 Non-Communicable Diseases and their Risk Factors	2013–2016	Yes	Narrative Response and Workplan	Yes

## Appendix 4. Alignment of NCD strategic plans with activities specified in the 2013–2020 global action plan

At the request of member states, the WHO released an updated technical annex to the Global Action Plan in 2017. As part of this annex, the WHO developed a list of 40 prioritized interventions targeting Objective 3 – reducing exposure to modifiable risk factors – and 37 interventions targeting Objective 4 – strengthening health systems to address NCDs. All of these interventions specifically target either the four behavioral risk factors (in the case of Objective 3) or the four main disease categories (Objective 4) of the 4-by-4 framework. Seventeen of the 37 interventions are highlighted as ‘best buys’ that are ‘most cost-effective and feasible for implementation;’ of these, 13 are intersectoral risk-reduction interventions and just four are health-sector interventions – one for primary prevention (HPV vaccination) and three for secondary prevention (drug therapy for patients with high of risk of heart attack, drug therapy for patients with moderate-to-high risk of heart attack, and cervical cancer screening with timely treatment of precancerous lesion).

In contrast to the general objectives, for which we find a general alignment between the Global Action Plan and policy interventions proposed by countries, the specific interventions highlighted by the revised technical annex are less frequently reflected in strategic plans. Those interventions targeting the modifiable risk factors are more common, with 10 of the 40 interventions found in at least half of the strategic plans (tobacco taxation, creation of smoke-free spaces, anti-tobacco media campaigns, tobacco cessation programs, alcohol taxation, prevention and treatment of alcohol abuse, mass media campaign on health diets, public awareness campaigns for physical activity, physical education at schools, and the creation of public spaces for exercise). However, of the 37 health sector interventions targeting prevention, care, and treatment of NCDs, only two are found in 50% or more of strategic plans. Both of these are aimed at primary prevention: lifestyle interventions to prevent diabetes and Hepatitis B vaccination. The only additional intervention to be found in more than one-quarter of strategic plans, HPV vaccination, is also aimed at primary prevention. Therapeutic interventions, such as drug therapy after heart attack or stroke, ACE inhibitors, beta blockers and diuretics for congestive heart failure, diabetic retinopathy screening, treatment for early stage breast and cervical cancers, and inhaled steroids for respiratory diseases were all rare, found in fewer than 20% of strategic plans (Figure 4A).

**Table 4A.** Percentage of plans specifying the policy goals policy recommendations included in the Global Action Plan for the prevention and control of NCDs 2013–2020^a^; n = 24.

**Objective 1: To raise the priority accorded to the prevention and control of noncommunicable diseases in global, regional and national agendas and internationally agreed development goals, through strengthened international cooperation and advocacy**	**100%**
Raise public and political awareness, understanding and practice about prevention and control of NCDs	100%
Integrate NCDs into the social and development agenda and poverty alleviation strategies	54%
Strengthen international cooperation for resource mobilization, capacity-building, health workforce training and exchange of information on lessons learnt and best practices	92%
Engage and mobilize civil society and the private sector as appropriate and strengthen international cooperation to support implementation of the action plan at global, regional and national levels	100%
**Objective 2: To strengthen national capacity, leadership, governance, multisectoral action and partnerships to accelerate country response for the prevention and control of noncommunicable diseases**	**96%**
Prioritize and increase, as needed, budgetary allocations for prevention and control of NCDs without prejudice to the sovereign right of nations to determine taxation and other policies	92%
Assess national capacity for prevention and control of NCDs	63%
Develop and implement a national multisectoral policy and plan for the prevention of control of NCDs through multi-stakeholder engagement	79%
**Objective 3: To reduce modifiable risk factors for noncommunicable diseases and underlying social determinants through creation of health-promoting environments**	**96%**
Strengthen the effective implementation of the WHO FCTC and its protocols	95%
Establish and operationalize national mechanisms for coordination of the WHO FCTC implementation as part of national strategy with specific mandate, responsibilities and resources	13%
*If not members, consider implementing the measures set out in the WHO FCTC and its protocols, as the foundational instrument in global tobacco control*	*100%^†^*
Implement the WHO Global strategy to reduce harmful use of alcohol through multisectoral actions in the recommended target areas	75%
Strengthen leadership and increase commitment and capacity to address the harmful use of alcohol	*8%*
Increase awareness and strengthen the knowledge base on the magnitude and nature of problems caused by harmful use of alcohol by awareness programs, operational research, improved monitoring and surveillance systems	*13%*
Implement the WHO Global Strategy on Diet, Physical Activity and Health	79%
Implement the WHO recommendations on the marketing of foods and non-alcoholic beverages to children	42%
**Objective 4: To strengthen and orient health systems to address the prevention and control of noncommunicable diseases and the underlying social determinants through people-centered primary health care and universal health coverage**	**100%**
Integrate very cost-effective noncommunicable disease interventions into the basic primary health care package with referral systems to all levels of care to advance the universal health coverage agenda	58%
Explore viable health financing mechanisms and innovative economic tools supported by evidence	42%
Scale up early detection and coverage, prioritizing very cost-effective high-impact interventions including cost-effective interventions to address behavioral risk factors	58%
Train the health workforce and strengthen capacity of health system particularly at primary care level to address the prevention and control of noncommunicable diseases	96%
Improve the availability of the affordable basic technologies and essential medicines, including generics, required to treat major noncommunicable diseases, in both public and private facilities	100%
Develop and implement a palliative care policy, including access to opioids analgesics for pain relief, together with training for health workers	33%
Expand the use of digital technologies to increase health service access and efficacy for NCD prevention, and to reduce the costs in health care delivery	8%
**Objective 5: To promote and support national capacity for high-quality research and development for the prevention and control of noncommunicable diseases**	**83%**
Develop and implement a prioritized national research agenda for noncommunicable diseases	63%
Prioritize budgetary allocation for research on noncommunicable disease prevention and control	42%
Strengthen human resources and institutional capacity for research	54%
Strengthen research capacity through cooperation with foreign and domestic research institutes	46%
**Objective 6: To monitor the trends and determinants of noncommunicable diseases and evaluate progress in their prevention and control**	**92%**
Develop national targets and indicators based on global monitoring framework and linked with a multisectoral policy and plan	38%
Strengthen human resources and institutional capacity for surveillance and monitoring and evaluation	67%
Establish and or strengthen a comprehensive noncommunicable disease surveillance system, including reliable registration of deaths by cause, cancer registration, periodic data collection on risk factors and monitoring national response	88%
Integrate noncommunicable disease surveillance and monitoring into national health information systems	75%

^a^Specific actions taken from the revised Appendix 2; 2017.

*^†^*N = 4 countries have not signed onto the FCTC: Côte d’Ivoire, Ethiopia, Malawi, and Sierra Leone.

## References

[1] Institute for Health Metrics and Evaluation (IHME). GBD Compare Seattle, WA: IHME, University of Washington; 2017 [cited 2019 410]. Available from: http://ihmeuw.org/54xd

[2] Bukhman G, Mocumbi AO, Horton R. Reframing NCDs and injuries for the poorest billion: a Lancet Commission. Lancet. 2015 9 26;386:1221–19. 26403914 10.1016/S0140-6736(15)00278-0

[3] Binagwaho A, Muhimpundu MA, Bukhman G, et al. 80 under 40 by 2020: an equity agenda for NCDs and injuries. Lancet. 2014 1 4;383:3–4. 24388297 10.1016/S0140-6736(13)62423-X

[4] Magnussen R. Non-communicable diseases and global health politics. In: McInnes C, Lee K, Youde J, editors. The Oxford handbook of global health politics. New York, NY: Oxford University Press; 2020. p. 626–659.

[5] Stanaway JD, Afshin A, Gakidou E, et al. Global, regional, and national comparative risk assessment of 84 behavioural, environmental and occupational, and metabolic risks or clusters of risks for 195 countries and territories, 1990–2017: a systematic analysis for the Global Burden of Disease Study 2017. Lancet. 2018;392:1923–1994. 30496105 10.1016/S0140-6736(18)32225-6 PMC6227755

[6] Foreman KJ, Marquez N, Dolgert A, et al. Forecasting life expectancy, years of life lost, and all-cause and cause-specific mortality for 250 causes of death: reference and alternative scenarios for 2016-40 for 195 countries and territories. Lancet. 2018 11 10;392:2052–2090. 30340847 10.1016/S0140-6736(18)31694-5 PMC6227505

[7] Institute for Health Metrics and Evaluation (IHME). GBD foresight | viz Hub. 2019 [cited 2019 825]. Available from: https://vizhub.healthdata.org/gbd-foresight/#

[8] Rani M, Nusrat S, Hawken LH. A qualitative study of governance of evolving response to non-communicable diseases in low-and middle- income countries: current status, risks and options. BMC Public Health. 2012 10 16;12:877. 23067232 10.1186/1471-2458-12-877 PMC3487912

[9] World Health Organization. 2008–2013 action plan for the global strategy for the prevention and control of noncommunicable diseases. Geneva: World Health Organization; 2008.

[10] General Assembly Resolution on the prevention and control of non-communicable diseases, A/RES/64/265 (2010).

[11] Scope and modalities of the comprehensive review and assessment of the progress achieved in the prevention and control of non-communicable diseases. New York, NY: General Assembly of the United Nations; 2014.

[12] Scope, modalities, format and organization of the third high-level meeting of the General Assembly on the prevention and control of non-communicable diseases. New York, NY: General Assembly of the United Nations; 2018.

[13] World Health Organization. Global action plan for the prevention and control of noncommunicable diseases 2013–2020. Geneva, Switzerland: World Health Organization; 2013.

[14] Bukhman G, Mocumbi AO, Atun R, et al. The Lancet NCDI Poverty Commission: bridging a gap in universal health coverage for the poorest billion. Lancet. 2020. (forthcoming). Available from: 10.1016/PII PMC7489932 32941823

[15] Puska P. Prevention of cardiovascular diseases: a spearhead for control of noncommunicable diseases. East Mediterr Health J. 2014 7 8;20:407–408. 25023766

[16] World Health Organization. Assessing national capacity for the prevention and control of noncommunicable diseases: report of the 2015 global survey. Geneva: World Health Organization; 2015.

[17] Ota MOC, Kirigia DG, Asamoah-Odei E, et al. Proceedings of the first African Health Forum: effective partnerships and intersectoral collaborations are critical for attainment of Universal Health Coverage in Africa. BMC Proc. 2018;12:8. 29997696 10.1186/s12919-018-0104-2 PMC6031170

[18] World Health Organization. Country planning cycle database. 2019 [cited 2019 129]. Available from: http://www.nationalplanningcycles.org

[19] World Health Organization. NCD Document Repository [Internet]. 2019 [cited 2019 Jan 29]. Available from: https://extranet.who. int/ncdccs/documents/.

[20] International Cancer Control Partnership. National plans portal [Internet]. 2019 [cited 2019 Jan 29]. Available from https://www.iccp-portal.org.

[21] Gale N, Heath G, Cameron E, et al. Using the framework method for the analysis of qualitative data in multi-disciplinary health research. BMC Med Res Methodol. 2013;13:117. 24047204 10.1186/1471-2288-13-117 PMC3848812

[22] Global Burden of Disease Collaborative Network. Global Burden of Disease Study 2015 (GBD 2015) cause, REI, and location hierarchies. Seattle, USA: Institute for Health Metrics and Evaluation (IHME); 2016.

[23] World Health Organization. Updated Appendix 3 of the WHO global NCD action plan 2013–2020. Geneva: World Health Organization; 2017.

[24] Ezzati M, Riboli E. Can noncommunicable diseases be prevented? Lessons from studies of populations and individuals. Science. 2012 9 21;337:1482–1487. 22997325 10.1126/science.1227001

[25] Virtanen M, Ervasti J, Head J, et al. Lifestyle factors and risk of sickness absence from work: a multicohort study. Lancet Public Health. 2018 11;3:e545–e554. 30409406 10.1016/S2468-2667(18)30201-9 PMC6220357

[26] World Health Organization, editor. Noncommunicable diseases: what gets measured, gets done; side-event on targets and indicators for NCDs. UN High-level Meeting on NCDs; 2011; New York.

[27] Magnusson RS, McGrady B, Gostin L, et al. Legal capacities required for prevention and control of noncommunicable diseases. Bull World Health Organ. 2019 2 1;97:108–117. 30728617 10.2471/BLT.18.213777 PMC6357565

[28] Buse K, Tanaka S, Hawkes S. Healthy people and healthy profits? Elaborating a conceptual framework for governing the commercial determinants of non-communicable diseases and identifying options for reducing risk exposure. Global Health. 2017 6 15;13:34. 28619031 10.1186/s12992-017-0255-3 PMC5472958

[29] Mohamed SF, Juma P, Asiki G, et al. Facilitators and barriers in the formulation and implementation of tobacco control policies in Kenya: a qualitative study. BMC Public Health. 2018 8 15;18:960. 30168390 10.1186/s12889-018-5830-x PMC6117633

[30] Juma K, Juma PA, Mohamed SF, et al. First Africa non-communicable disease research conference 2017: sharing evidence and identifying research priorities. J Glob Health. 2019 6;8:020301. 30774938 10.7189/jogh.09.010201 PMC6370979

[31] Juma PA, Mohamed SF, Matanje Mwagomba BL, et al. Non-communicable disease prevention policy process in five African countries. BMC Public Health. 2018 8 15;18:961. 30168393 10.1186/s12889-018-5825-7 PMC6117619

[32] Juma PA, Mapa-Tassou C, Mohamed SF, et al. Multi-sectoral action in non-communicable disease prevention policy development in five African countries. BMC Public Health. 2018 8 15;18:953. 30168391 10.1186/s12889-018-5826-6 PMC6117629

[33] Lupafya PC, Mwagomba BL, Hosig K, et al. Implementation of policies and strategies for control of noncommunicable diseases in Malawi: challenges and opportunities. Health Educ Behav. 2016 4;43:64S–9S. 27037149 10.1177/1090198115614313

[34] Siddharthan T, Ramaiya K, Yonga G, et al. Noncommunicable diseases in East Africa: assessing the gaps in care and identifying opportunities for improvement. Health Aff (Millwood). 2015 9;34:1506–1513. 26355052 10.1377/hlthaff.2015.0382 PMC4568565

[35] Shiroya V, Neuhann F, Muller O, et al. Challenges in policy reforms for non-communicable diseases: the case of diabetes in Kenya. Glob Health Action. 2019;12:1611243. 31117896 10.1080/16549716.2019.1611243 PMC6534207

[36] Mukanu MM, Zulu JM, Mweemba C, et al. Responding to non-communicable diseases in Zambia: a policy analysis. Health Res Policy Syst. 2017 4 24;15:34. 28438177 10.1186/s12961-017-0195-7 PMC5402674

[37] Witter S, Zou G, Diaconu K, et al. Opportunities and challenges for delivering non-communicable disease management and services in fragile and post-conflict settings: perceptions of policy-makers and health providers in Sierra Leone. Confl Health. 2020;14:3. 31921333 10.1186/s13031-019-0248-3 PMC6945746

[38] Nyaaba GN, Stronks K, De-graft Aikins A, et al. Tracing Africa’s progress towards implementing the non-communicable diseases global action plan 2013–2020: a synthesis of WHO country profile reports. BMC Public Health. 2017 4 5;17:297. 28381252 10.1186/s12889-017-4199-6 PMC5382491

[39] Mamka Anyona R, de Courten M. An analysis of the policy environment surrounding noncommunicable diseases risk factor surveillance in Kenya. AIMS Public Health. 2014;1:256–274. 29546090 10.3934/publichealth.2014.4.256 PMC5690257

[40] Schwartz J, Guwatudde D, Nugent R, et al. Looking at non-communicable diseases in Uganda through a local lens: an analysis using locally derived data. Global Health. 2014;10:77. 25406738 10.1186/s12992-014-0077-5 PMC4240853

[41] Lemieux-Charles L, McGuire WL. What do we know about health care team effectiveness? A review of the literature. Med Care Res Rev. 2006 6;63:263–300. 16651394 10.1177/1077558706287003

[42] Feinerer I, Hornik K, Meyer D. Text mining infrastructure in R. J Stat Softw. 2008;25:1–53.

